# The Association of Women’s Empowerment with HIV-Related Indicators: A Pooled Analysis of Demographic and Health Surveys in Sub-Saharan Africa

**DOI:** 10.1007/s44197-023-00153-w

**Published:** 2023-09-26

**Authors:** Theresa Schierl, Luana Fiengo Tanaka, Stefanie J. Klug, Andrea Sylvia Winkler, Dominik Stelzle

**Affiliations:** 1 Center for Global Health, Department of Neurology, Technical University of Munich, Ismaninger Str. 22, 81675 Munich, Germany; 2 Department of Sport and Health Sciences, Technical University of Munich, Georg Brauchle Ring 60/62, Munich, Germany; 3 Center for International Health, Ludwig Maximilian University of Munich (LMU), Munich, Germany; 4 Centre for Global Health, Institute of Health and Society, University of Oslo, Oslo, Norway

**Keywords:** Women’s empowerment, HIV testing, Demographic and Health Surveys, Sub-Saharan Africa, sexual health, reproductive health

## Abstract

**Background:**

Women’s empowerment is an important factor for HIV prevention, but the association with HIV-related indicators has never been quantified. In this study, we examined the association between women’s empowerment and selected HIV-related indicators.

**Methods:**

We used the latest Demographic and Health Surveys that included HIV testing among other biomarkers of 31 countries in sub-Saharan Africa. Empowerment was measured by the Survey-based Women’s EmPowERment (SWPER) index and was compared to the HIV-related indicators: HIV status, HIV testing (ever and in the past 12 months), condom use at last high-risk sex, the ability to ask the partner to use a condom, and the ability to refuse sex.

**Results:**

208,947 women were included in the analysis, of whom 100,924 (48%) were considered highly empowered and 21,933 (10%) as lowly empowered. There was no association between empowerment and HIV status (OR = 1.12, 95% confidence interval [CI] 0.98–1.28). Highly empowered women were more likely to have ever been tested for HIV (OR = 1.67, 95% CI 1.60–1.74) but less likely to have been tested for HIV in the past 12 months (OR = 0.92, 95%CI 0.88–0.96). Highly empowered women were more commonly able to ask the partner to use a condom (OR = 1.69, 95% CI 1.63–1.75) and to refuse sex (OR = 1.78, 95%CI 1.72–1.85).

**Conclusions:**

Women’s empowerment does not seem to be linked to HIV status, but it is strongly associated with a woman’s ability to make decisions about their sexual behavior. Empowering women and young girls has the potential to contribute toward achieving the United Nations’ goal of ending AIDS by 2030.

**Supplementary Information:**

The online version contains supplementary material available at 10.1007/s44197-023-00153-w.

## Background

In sub-Saharan Africa (SSA), women are disproportionately affected by HIV: 64% of the 23.8 million living with HIV in 2020 were women. Disparities are similarly pronounced in the younger population globally: 2.4 million young women (15–24 years) are living with HIV, which equals 61 percent of all young people living with HIV [1], and despite comprising 10% of the population in SSA, young women account for 20% of new HIV infections in 2018 [2]. Globally, in 2016, there were an estimated 2.4 million adolescent girls and young women living with HIV, that constitute 61 per cent of all young people living with HIV [15–24].

Although some progress has been achieved since the early days of the HIV epidemic in the region, the gap in the HIV burden between men and women still persists [1, 3]. Reasons for these disparities include limited or lack of education, insufficient knowledge about HIV, poor access to health services, intimate partner violence, child marriage, and financial dependence, among others [1]. These disparities are strengthened by the general imbalance in power between men and women. Evidence suggests that the unequal levels of power between men and women might lead to a lack of agency especially when it comes to negotiating reproductive health [4, 5]. Connell’s theory of gender and power further explains the impact of socially constructed gender roles on power dynamics within society, which in turn affects women’s agency. Wingood and colleagues applied Connell’s theory of gender and power to HIV prevention, recognizing that HIV disproportionately affects women, particularly in resource-limited settings and sought to address the gender-based factors contributing to women’s vulnerability to HIV infection. This framework for developing interventions that target gender-based factors contributing to women’s vulnerability to HIV suggests that interventions may include promoting gender equality, women’s empowerment and challenging harmful gender norms and stereotypes [6]. Empowerment is a multidimensional concept for which multiple definitions exist [7–9]. Empowerment is “the process of enhancing an individual’s or group’s capacity to make purposive choices and to transform those choices into desired actions and outcomes” as the World Bank defines [10]. For women, this can only happen if they can envision themselves as able and entitled to make decisions over their own life [11]. Further, the development of a critical view on women’s rights and gendered power relations is crucial in overcoming gender inequity [12].

Ewerling et al. developed the survey-based Women’s emPowERment index (SWPER) using individual data stemming from the Demographic and Health Surveys (DHS) and based on 15 variables, grouped into three dimensions of empowerment. The index was first validated for SSA [8], and later extended to low- and middle-income countries globally [13]. The SWPER Index has been used to assess the association between empowerment and health-related outcomes [13, 14], but to date, HIV-related factors have not been examined, although previous studies have employed other less robust measurements of women’s empowerment and compared them with HIV-related factors [15–17]. That is why this study investigated the association between women’s empowerment, as measured by the SWPER index, and different HIV-related indicators in SSA. This study may help to define whether the SWPER index is a useful tool to monitor HIV-related factors associated with women’s empowerment.

## Methods

### Data Source

The DHS are nationally representative household surveys conducted in over 90 countries, preferably every 5 years. A country’s sample size ranges from 5000 to 30,000 households and collects indicators on population, health, and data collection is done via validated questionnaires in randomly selected households and consists of four parts: The household questionnaire (collecting key characteristics of each household); the women’s and men’s questionnaires, respectively, collect data through individual interviews; lastly, the biomarker questionnaire which is only administered to a random subset and collects data about anthropometric measurements and levels of hemoglobin, as well as tests for different biomarkers, such as HIV [18]. The questionnaires cover topics such as domestic violence, education, family planning, HIV/AIDS, women’s empowerment, among others.

For this study, we analyzed data from 31 countries, which had conducted surveys between 2008 and 2018, for which HIV testing results were available (Table 1).

**Table 1:** Socio-demographic characteristics of included participants by empowerment category (decision-making), Demographic and Health Surveys in sub-Saharan Africa

	Survey Year	Empowerment			Total
		Low	Medium	High	
*Country*					
Angola	2015/16	80 (1%)	1153 (17%)	5641 (82%)	6874
Burkina Faso	2010	1589 (13%)	8272 (68%)	2335 (19%)	12 196
Benin	2017/18	568 (6%)	4807 (49%)	4534 (46%)	9909
Burundi	2016/17	1411 (15%)	2376 (25%)	5580 (60%)	9367
Democratic Republic of the Congo	2013/14	248 (2%)	5082 (47%)	5484 (51%)	10 815
Cote d'Ivoire	2011/12	577 (11%)	2932 (56%)	1716 (33%)	5224
Cameroon	2018	488 (7%)	2524 (37%)	3872 (56%)	6884
Ethiopia	2016	1051 (11%)	1708 (18%)	6479 (70%)	9237
Gabon	2012	58 (2%)	925 (28%)	2347 (70%)	3330
Ghana	2014	63 (1%)	990 (21%)	3753 (78%)	4805
Gambia	2013	273 (5%)	2212 (39%)	3140 (56%)	5625
Guinea	2018	987 (15%)	3188 (47%)	2622 (39%)	6797
Kenya	2008/09	581 (13%)	1582 (35%)	2408 (53%)	4571
Comoros	2012	181 (8%)	1022 (45%)	1053 (47%)	2256
Lesotho	2014	58 (2%)	773 (28%)	1904 (70%)	2735
Mali	2012/13	1935 (24%)	5042 (63%)	1044 (13%)	8021
Malawi	2015/16	2285 (16%)	5373 (37%)	7001 (48%)	14 660
Niger	2012	1107 (13%)	5603 (66%)	1739 (21%)	8449
Namibia	2013	156 (6%)	472 (18%)	1992 (76%)	2620
Rwanda	2014/15	543 (8%)	1731 (26%)	4265 (65%)	6538
Sierra Leone	2013	697 (7%)	3762 (39%)	5256 (54%)	9715
Senegal	2017	1366 (20%)	4048 (58%)	1581 (23%)	6995
Sao Tome and Principe	2008/09	11 (1%)	478 (30%)	1087 (69%)	1575
Eswatini	2006/07	324 (18%)	755 (43%)	692 (39%)	1771
Chad	2014/15	853 (8%)	7095 (63%)	3240 (29%)	11 187
Togo	2013/14	291 (5%)	2854 (51%)	2495 (44%)	5640
Tanzania	2015/16	1781 (31%)	2157 (37%)	1829 (32%)	5767
Uganda	2016	1398 (14%)	3431 (34%)	5240 (52%)	10 069
South Africa	2016	62 (2%)	258 (10%)	2389 (88%)	2709
Zambia	2018	729 (10%)	2190 (31%)	4087 (58%)	7007
Zimbabwe	2015	183 (3%)	1298 (23%)	4120 (74%)	5601
*Region*					
Eastern and southern Africa		10 560 (13%)	24 104 (29%)	47 986 (58%)	82 650
Western and central Africa		11 373 (9%)	61 986 (49%)	52 938 (42%)	126 297
*Age group (years)*					
15–19		772 (9%)	4824 (54%)	3390 (38%)	8986
20–24		3338 (10%)	16 048 (47%)	14 874 (43%)	34 261
25–29		4581 (10%)	19 871 (43%)	21 649 (47%)	46 102
30–34		4455 (11%)	16 741 (40%)	20 443 (49%)	41 640
35–39		3861 (11%)	13 009 (38%)	17 469 (51%)	34 339
40–44		2762 (11%)	9053 (36%)	13 091 (53%)	24 906
45–49		2163 (12%)	6543 (35%)	10 008 (53%)	18 715
*Residence*					
Urban		3846 (6%)	22 985 (36%)	36 792 (58%)	63 624
Rural		18 087 (12%)	63 105 (43%)	64 132 (44%)	14 5324
*Education*					
No education		13 671 (15%)	45 242 (49%)	33 128 (36%)	92 041
Primary		6809 (10%)	25 928 (38%)	34 724 (51%)	67 461
Secondary		1353 (3%)	13 608 (32%)	27 909 (65%)	42 870
Higher		101 (2%)	1311 (20%)	5163 (79%)	6575
*Wealth*					
Lowest quintile		5469 (13%)	18 082 (44%)	17 571 (43%)	41 123
Low		5084 (12%)	18 482 (43%)	19 214 (45%)	42 779
Medium quintile		4700 (11%)	17 526 (42%)	19 827 (47%)	42 053
Higher		4011 (10%)	17 446 (41%)	20 751 (49%)	42 208
Highest quintile		2669 (7%)	14 554 (36%)	23 561 (58%)	40 785
*HIV (biomarker)*					
Test negative		8358 (10%)	34 163 (40%)	43 299 (50%)	85 820
Test positive		259 (6%)	1302 (30%)	2758 (64%)	4319
No HIV test		13 316 (11%)	50 625 (43%)	54 868 (46%)	118 808
*Sexual activity in the last 12 months*					
No		3670 (17%)	13 995 (16%)	12 958 (13%)	30 623
Yes		18 257 (83%)	72 028 (84%)	87 875 (87%)	178 160
*Non-regular sexual partner*					
No		21 757 (99%)	85 209 (99%)	99 021 (98%)	205 987
Yes		176 (1%)	881 (1%)	1903 (2%)	2960
**Overall**		**21 933 (10%)**	**86 090 (41%)**	**100 924 (48%)**	**208 947**

### Assessment of Women’s Empowerment

We used the SWPER index to assess women’s empowerment. The SWPER index is based on DHS data and is comprised of 15 selected items that are grouped into three dimensions of empowerment, of which all include all 15 items with different weighting, the dimensions are: attitudes towards violence, social independence and decision-making [8]. The questions and formulas for computing the SWPER have previously been published, they can also be found in the appendix [8].

Empowerment levels were standardized according to the SSA region (eastern and southern Africa and western and central Africa) and categorized into low, medium, and high empowerment according to the definition by Ewerling et al. [13]. We analyzed all three dimensions but focused on the decision-making category. We selected decision-making as the key dimension for our analyses, as it puts most of the weight on household-level decisions, rather than at the societal level and especially gives relevance to the item “who makes decisions about the respondent’s health”, which is crucial for HIV prevention. All items are used to calculate all three empowerment components, the scores are calculated using different weights for the different items.

### Selected Outcomes

We analyzed the association between women’s empowerment and six outcomes: HIV status (positive/negative), HIV testing (ever tested among those who had ever had sex and tested in the past 12 months among those who had sex in the past 12 months), condom use at last high-risk sex (i.e., sex with a non-regular partner), a woman’s ability to refuse sex, and the ability to ask a partner to use a condom. We used the UNAIDS indicator definitions for the HIV testing variables and the condom use at the last high-risk sex variable [19].

### Statistical Analyses

We present empowerment levels for each country as total weighted cases and proportions. The association between empowerment level and the outcome variables was examined using logistic regression models raw and adjusted for age (5 year age groups), type of residence (urban vs rural), and wealth quintile, having low empowerment as the reference category. All models included a fixed-effect for countries. Odds ratios (OR) are presented with 95% confidence intervals (95% CI). For each of the six outcome variables, analyses were furthermore stratified by type of residence, region, and age group. When applicable, results were also presented for the subgroup of HIV negative women (i.e., all indicators apart from HIV status). We conducted a complete case analysis, considering that the analyzed data are missing completely at random, which was previously reported [14]. All statistical analyses were performed using R (version 4.0.2), using the ‘survey’ package [20].

### Ethics and Consent

The questionnaires used by the DHS undergo approval by ICF Institutional Review Board (IRB). Country-specific procedures are evaluated by a local IRB. The informed consent statement is read to all potential participants, before data/sample collection, who can accept or refuse to join the survey.

## Results

### Characteristics of the Study Population

Overall, 404,921 women living in 31 countries in SSA were included in the DHS. Among these, 208,947 women had information available for calculating the SWPER index. 126,297 (60.4%) women were from 17 countries in west/central Africa (WCA) and 82,650 (39.6%) from 14 countries in east/south Africa (ESA) (Table 1).

The median age was 27 years (interquartile range from 20 to 36 years), most women were living in rural areas (69.4%), and a high proportion had no formal education (44.0%). 89,139 had an HIV test performed, of whom 4319 (4.8%) were living with HIV. Most women (85%) reported being sexually active in the last 12 months, and 0.9% reported a non-regular partner in this period.

**Fig. 1:**
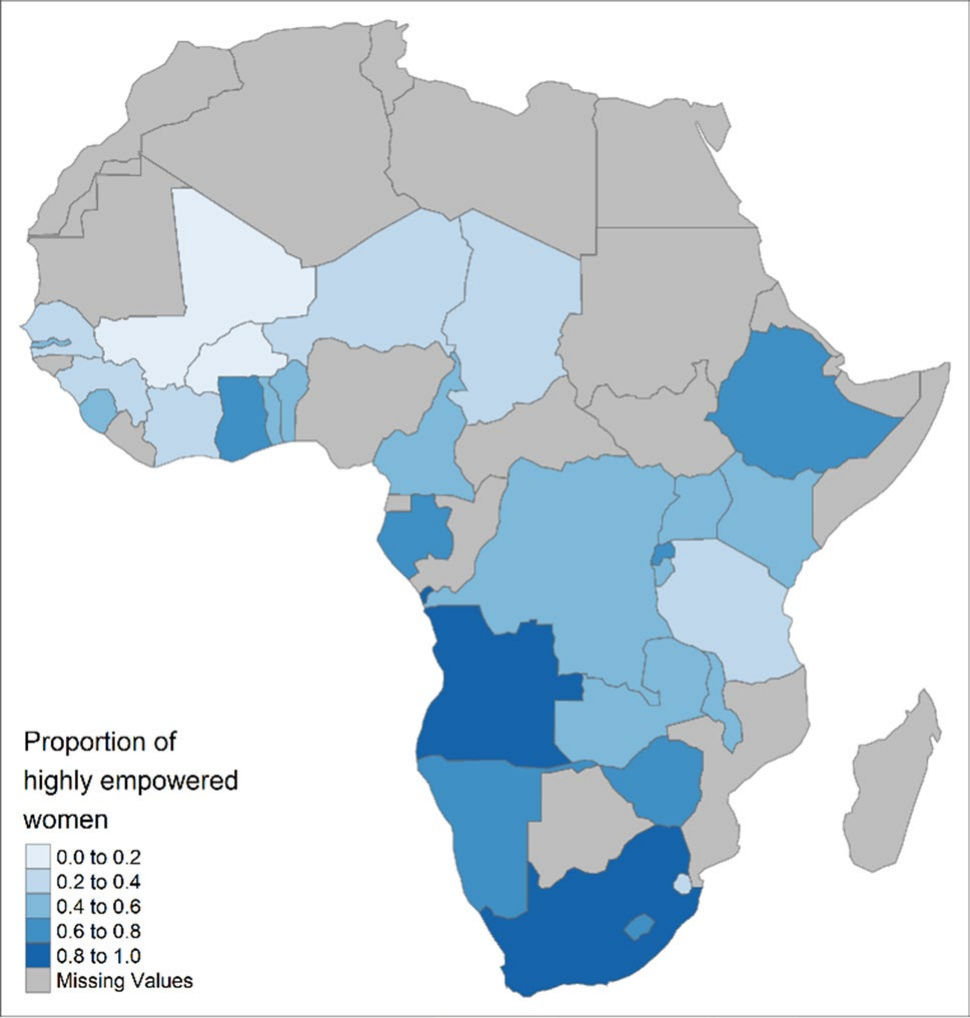
Proportion of highly empowered women, by country in sub-Saharan Africa

Overall, 48% of women were highly empowered and 10% lowly empowered, but empowerment levels varied greatly across countries. More women in ESA (58%) were highly empowered than in WCA (42%) (Fig. 1). The countries with the largest proportion of highly empowered women were South Africa (88%), Angola (82%), Ghana (78%), Namibia (76%) and Zimbabwe (74%), whereas Mali (13%), Burkina Faso (19%), Niger (21%), Senegal (23%), and Chad (29%) had the lowest proportions (Table 1). Women living in urban areas (58 vs 46% rural) and women with higher education (79 vs 36% with no education) were more empowered.

### Distribution of the Outcome Variables

About 5% of women were living with HIV, the majority (58%) had been tested at least once and in the past 12 months (53%), were able to refuse sex (62%) and to ask a partner to use a condom (54%) (Table 2). When examining these variables by empowerment level, 6% of highly empowered women were living with HIV (versus 3% among lowly empowered), 67% had been tested for HIV (versus 51% among lowly empowered), 55% having been tested in the past 12 months (versus 52% among lowly empowered). Thirty-five percent of medium and 33% of highly empowered women used a condom at last high-risk sex (versus 23% among low). Most highly empowered women were able to refuse sex (69% versus 48% low) and were able to ask partner to use a condom (63% versus 42% low).

**Table 2:** Distribution of HIV status and prevention variables by empowerment status (decision-making), Demographic and Health Surveys in sub-Saharan Africa

	Empowerment (decision-making)			Overall
	Low	Medium	High	
HIV status: positive	264/8648 (3%)	1321/35 891 (4%)	2791/47 898 (6%)	4376/92 437 (5%)
Ever tested for HIV	11 077/21 838 (51%)	41 481/85 609 (48%)	67 383/10 0542 (67%)	119 941/207 989 (58%)
HIV test in the past 12 months	4977/9501 (52%)	17 795/35 307 (50%)	32 570/59 405 (55%)	55 342/104 213 (53%)
Condom use at last high-risk sex	41/176 (23%)	310/881 (35%)	620/1903 (33%)	971/2960 (33%)
Ability to refuse sex	9460/19 658 (48%)	41 644/74 634 (56%)	63 222/91 428 (69%)	114 326/185 720 (62%)
Ability to ask partner to use a condom	8093/19 319 (42%)	34 049/73 309 (46%)	56 262/89 986 (63%)	98 404/182 614 (54%)

### Association Between Empowerment Level and Outcome Variables

**Fig. 2:**
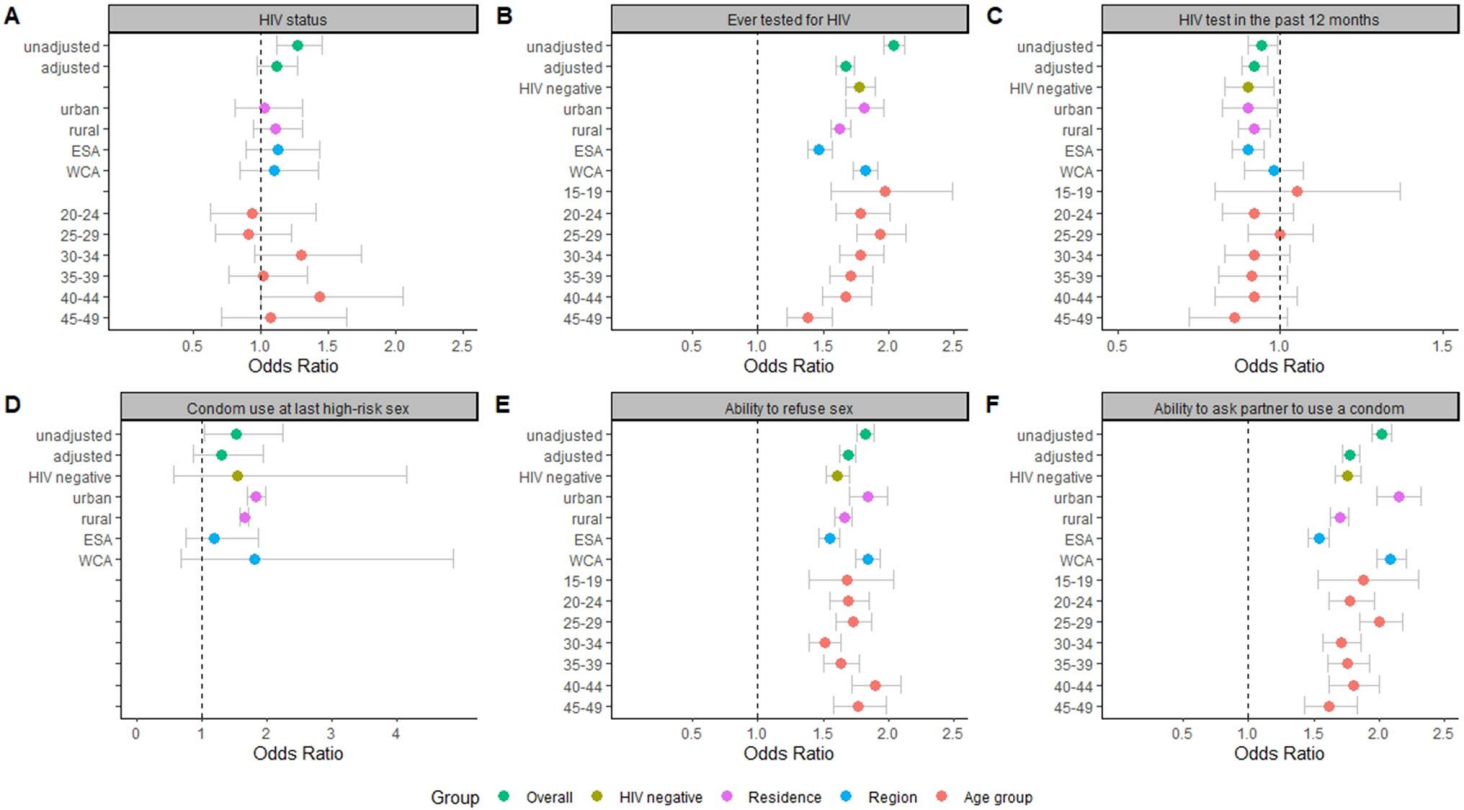
Association between high versus low empowerment (decision-making) and outcome variables. The figure shows the odds ratios including 95% confidence intervals between high empowerment and low empowerment for six different factors. HIV status means HIV status positive; *ESA* Eastern and southern Africa; *WCA* Western and central Africa

Table 3 displays the odds ratios of medium and high versus low empowerment for the selected outcomes. In the unadjusted model, high empowerment was associated with HIV status (OR = 1.28, 95%CI 1.12–1.46 high vs low empowerment). After adjustment, however, HIV status was no longer associated with empowerment (adjusted OR (aOR) = 1.12, 95%CI 0.98–1.28 high vs low empowerment; aOR = 1.06, 95%CI 0.92–1.22 for medium vs low empowerment), nor with the other variables (Fig. 2A).

**Table 3:** Association between empowerment level (decision-making) and outcome variables, Demographic and Health Surveys in sub-Saharan Africa, 2006–2018

Variable	Empowerment level (decision-making)	OR (95%CI) unadjusted	OR (95%CI) Adjusted^a^	OR (95%CI) HIV negative^b^	OR (95%CI) ESA	OR (95%CI) WCA
HIV status: positive	Low	Reference	Reference	NA	Reference	Reference
	Medium	1.14 (1.00–1.31)	1.06 (0.92–1.22)	NA	1.08 (0.92–1.28)	1.02 (0.79–1.32)
	High	1.28 (1.12–1.46)	1.12 (0.98–1.28)	NA	1.13 (0.97–1.32)	1.10 (0.85–1.43)
Ever tested for HIV	Low	Reference	Reference	Reference	Reference	Reference
	Medium	1.52 (1.46–1.58)	1.38 (1.33–1.44)	1.46 (1.37–1.56)	1.43 (1.34–1.54)	1.43 (1.35–1.50)
	High	2.04 (1.96–2.12)	1.67 (1.60–1.74)	1.78 (1.67–1.90)	1.47 (1.38–1.57)	1.82 (1.73–1.92)
HIV test in the past 12 months	Low	Reference	Reference	Reference	Reference	Reference
	Medium	0.98 (0.93–1.03)	0.96 (0.92–1.01)	0.95 (0.87–1.03)	0.96 (0.90–1.01)	1.01 (0.92–1.11)
	High	0.94 (0.90–0.99)	0.92 (0.88–0.96)	0.90 (0.83–0.98)	0.9 (0.85–0.95)	0.98 (0.89–1.07)
Condom use at last high-risk sex	Low	Reference	Reference	Reference	Reference	Reference
	Medium	1.87 (1.25–2.8)	1.69 (1.12–2.55)	2.39 (0.87–6.56)	1.76 (1.1–2.81)	2.13 (0.79–5.75)
	High	1.53 (1.03–2.26)	1.31 (0.87–1.95)	1.54 (0.57–4.16)	1.19 (0.76–1.86)	1.81 (0.68–4.87)
Ability to refuse sex	Low	Reference	Reference	Reference	Reference	Reference
	Medium	1.39 (1.34–1.44)	1.34 (1.29–1.39)	1.33 (1.26–1.40)	1.31 (1.24–1.38)	1.40 (1.33–1.47)
	High	1.82 (1.76–1.89)	1.69 (1.63–1.75)	1.61 (1.52–1.70)	1.55 (1.47–1.63)	1.84 (1.75–1.94)
Ability to ask partner to use a condom	Low	Reference	Reference	Reference	Reference	Reference
	Medium	1.48 (1.43–1.54)	1.39 (1.34–1.45)	1.40 (1.32–1.49)	1.44 (1.36–1.52)	1.48 (1.40–1.56)
	High	2.02 (1.95–2.10)	1.78 (1.72–1.85)	1.76 (1.66–1.86)	1.54 (1.46–1.62)	2.09 (1.98–2.21)

^a^Adjusted for country, age, place of residence and wealth quintile

^b^Subset of HIV negative women (tested as part of the biomarker studies within the Demographic and Health Surveys)

*ESA* Eastern and southern Africa; *WCA* western and central Africa; *NA* not applicable

We detected a strong association between ever been tested for HIV and empowerment level (aOR = 1.67, 95%CI 1.60–1.74 for high versus low empowerment, aOR = 1.38, 95%CI 1.33–1.44 for medium versus low empowerment). The effect of empowerment on HIV testing did not differ between ESA and WSA, was not different for the subgroup of HIV negative women compared with all women. The effect of empowerment on HIV testing decreased with increasing age (Fig. 2B).

For HIV testing in the past 12 months, however, highly empowered women had lower odds of having been tested in the past 12 months than lowly empowered women (aOR = 0.92, 95%CI 0.88–0.96). This association was also found in the subgroup of HIV negative women and for women in ESA, but not for those in WCA (Fig. 2C).

An association between empowerment levels and condom use at last high-risk sex was seen in the unadjusted models but only retained statistically significant for medium versus low empowerment (aOR = 1.69, 95%CI 1.12–2.55, Table 3, Fig. 2D).

We observed strong associations between empowerment and the ability to refuse sex (aOR = 1.69, 95%CI 1.63–1.75 for high versus low empowerment, aOR = 1.34, 95%CI 1.29–1.39 for medium versus low empowerment) and the ability to ask the partner to use a condom (aOR = 1.78, 95%CI 1.72–1.85 for high versus low, aOR = 1.39, 95%CI 1.34–1.45 for medium versus low). These associations were stronger in urban areas and in WCA and were significant among HIV negative women, but they were not associated with a woman’s age (Fig. 2E and F).

## Discussion

This study examined the association between women’s empowerment (primarily measured as the ability to make decisions) and HIV-related indicators in sub-Saharan Africa, based on DHS data conducted in 31 countries. Our results reveal that highly empowered women are more likely to have ever been tested for HIV, being able to ask their partner to use a condom and to refuse sex, some of the key factors in HIV prevention. Nevertheless, empowerment was not associated with HIV status, nor with HIV testing in the past 12 months and condom use at last high-risk sex.

Our primary finding that empowerment is linked to (ever) undergoing HIV testing, as demonstrated in the pooled analyses as well as in all countries except for six, aligns with prior studies that employed alternative measures of women’s empowerment or variables related to women’s empowerment in Africa [16, 21, 22] and other regions [23]. The SWPER index contains a question that significantly influences the decision-making dimension: “Who usually decides on respondent's health care?” [8]. As a result, if women can make decisions about their healthcare, it is likely that this includes the decision to undergo HIV testing. On the contrary, a similar association could not be identified for HIV testing in the past 12 months. Yaya et al. [15] in their analyses including DHS data from 33 SSA countries and using a specific question from DHS as a proxy for women’s empowerment reported similar null results [15]. The discrepancy between ever HIV testing and HIV testing in the past 12 months could arise from different reasons. First, these analyses were restricted to women who were sexually active in this period, therefore representing a subset of 85% of the population included in the other analysis. Also, pregnancy and visiting antenatal care may have resulted in HIV tests independent from empowerment. Moreover, this indicator could reflect women’s perception about their risk of acquiring HIV, which in turn is influenced by their partnership status and sexual behavior (condom use, known status of sexual partner) [24].

Empowerment was also not associated with HIV status. This might be due to the complexity of HIV transmission networks within sub-Saharan Africa, e.g., there are no questions on potential mother-to-child transmission included in DHS, which may have influenced our results [28].

Highly empowered women are more likely to make key decisions about their sexual behavior, including asking their partner to use a condom and refusing sex. Thus, having more control over their risk of acquiring HIV. An association between autonomy in household decision-making has been described and safer sex negotiation has been previously described in SSA [29].

Younger women (aged 15–19 years) are less likely to have ever been tested for HIV, this could be due to them not yet being in the age group targeted by large-scale HIV testing programs, as their HIV prevalence is also relatively low at overall less than 2%.

## Limitations

This study has limitations relating primarily to DHS design. Firstly, only ever partnered women aged 15–49 years were included in our analyses because most of the questions comprising the SWPER index are solely posed to this group within the DHS. Therefore, our findings are limited to this subset of women, and it is unclear if similar patterns would have been found among never partnered women, predominantly younger women and/or older women (50+ years). Furthermore, the use of modern technologies or ownership of modern technologies is not represented in any of the variables because these questions have been only recently included in the DHS questionnaires [15]. Including them would have added another aspect of empowerment improving the index further, but then the SWPER could have been calculated only for a few countries [8]. The sensitive nature of the questions could have led to potential biases. However, we believe this did not affect our analysis, as the DHS program ensures high methodological standards, including specialized interviewer training and privacy measures during interviews. Respondents are also provided with information and referrals for services related to domestic violence if needed [30].

Further limitations stem from using questionnaires to collect data. Although DHS tools are validated [28], language barriers and social desirability bias [29] may have impacted our results. Moreover, some SWPER index variables may reflect women's opinions rather than their realities. Lastly, several countries in the model have relatively low overall HIV prevalence, so measures to reduce infection and increase awareness of HIV transmission might not be a priority.

## Conclusions

HIV prevention programs often focus on women and young girls as they are particularly affected by HIV and are an important group to achieve the UN’s goal of ending AIDS by 2030 [3]. Our analyses represent the household level of a woman’s reality rather than the larger societal circumstances. HIV prevention and health literacy programs may aim at increasing women’s empowerment and in turn their agency over their health decisions, which may lead to fewer infections, earlier treatment initiation, improved survival as well as improved quality of life.

A large-scale social paradigm shift is needed to truly empower women and enable them to obtain the power within their relationships to make decisions about themselves and their reproductive health.

### Supplementary Information

Below is the link to the electronic supplementary material.

Supplementary file1 (PDF 72 kb)

## Data Availability

Data are available for download from https://dhsprogram.com/data/available-datasets.cfm.

## Abbreviations

AIDS: Acquired immunodeficiency syndrome
DHS: Demographic and Health Survey
ESA: East and South Africa
HIV: Human immunodeficiency virus
SSA: Sub-Saharan Africa
SWPER: Survey-based Women’s empowerment index
WCA: West and Central Africa
