# Supplementary material for: The Association of Women’s Empowerment with HIV-Related Indicators: A Pooled Analysis of Demographic and Health Surveys in Sub-Saharan Africa
Source: J Epidemiol Glob Health. 2023 Sep 26;13(4):816–24. doi: 10.1007/s44197-023-00153-w (PMC10686950; doi:10.1007/s44197-023-00153-w)
Supplement: Supplementary file 1 — Supplementary file1 (PDF 72 kb) [file 44197_2023_153_MOESM1_ESM.pdf]

Appendix Women's Empowerment and HIV in sub-Saharan Africa

The Survey-based Women's Empowerment Index (SWPER) developed by Ewerling et al. (2017) uses the following variables from the Demographic and Health Surveys by U.S.AID.[1]

• Beating not justified if wife goes out without telling husband
• Beating not justified if wife neglects the children
• Beating not justified if wife argues with husband
• Beating not justified if wife refuses to have sex with husband
• Beating not justified if wife burns the food
• Frequency of reading newspaper or magazine
• Woman's education in completed years of schooling
• Age of woman at first birth
• Age at first cohabitation
• Age difference: woman's age minus husband's age
• Education difference: woman's minus husband's years of schooling
• Who usually decides on respondent's health care
• Who usually decides on large household purchases
• Who usually decides on visits to family or relatives
• Respondent worked in past 12 months (this variable was excluded from the up-dated global version of the SWPER, as it was deemed too simplistic, as it does not reflect whether the women were working because they wanted to or they were forced, it is also unclear whether the women were paid for their work)

The variables above are summarized as three main categories influencing women's empowerment according to the SWPER Index: Attitudes towards violence, Social Independence and Decision making.

Calculation of the SWPER scores:

Attitudes towards Violence_score =
((-0.950) + (0.489*beat1) + (0.493*beat2) + (0.501*beat3) + (0.493*beat4) + (0.546*beat5) + (0.056*read)+(0.015*educ) + (-0.008*age1birth) + (-0.004*age1co-hab) + (0.002*age_diff) + (-0.004*educ_diff) + (0.008*decide1) + (-0.034*decide2) + (0.008*decide3) + (-0.001*work))/1.818

Social Independence_score = ((-5.360)+(-0.006*beat1) + (-0.020*beat2) + (0.000*beat3) + (0.000*beat4) + (0.003*beat5) + (0.549*read) + (0.09*educ) + (0.141*age1birth) + (0.131*age1cohab) + (0.026*age_diff) + (0.050*educ_diff) + (0.004*decide1) + (-0.013*decide2) + (-0.052*decide3)+(-0.060*work)) /1.475

Decision Making_score=((0.857) + (-0.001*beat1) + (-0.040*beat2) + (0.007*beat3) + (0.026*beat4) + (-0.014*beat5) + (0.150*read) + (0.026*educ) + (-0.019*age1birth) + (-0.006*age1cohab) + (0.012*age_diff) + (-0.009*educ_diff) + (0.770*decide1) + (0.831*decide2) + (0.768*decide3) + (0.180*work))/1.417

References

1. Ewerling F, Lynch JW, Victora CG, van Eerdewijk A, Tyszler M, Barros AJD. The SWPER index for women's empowerment in Africa: development and validation of an index based on survey data. *The Lancet. Global health.* Sep 2017;5(9):e916-e923.
